# Functional Characterisation of New Sesquiterpene Synthase from the Malaysian Herbal Plant, *Polygonum Minus*

**DOI:** 10.3390/molecules23061370

**Published:** 2018-06-04

**Authors:** Nor Azizun Rusdi, Hoe-Han Goh, Suriana Sabri, Ahmad Bazli Ramzi, Normah Mohd Noor, Syarul Nataqain Baharum

**Affiliations:** 1 Institute of Systems Biology (INBIOSIS), Universiti Kebangsaan Malaysia, Bangi 43600 UKM, Selangor, Malaysia; azizun@ums.edu.my (N.A.R.); gohhh@ukm.edu.my (H-H.G.); bazliramzi@ukm.edu.my (A.B.R.); normah@ukm.edu.my (N.M.N.); 2 Institutes for Tropical Biology and Conservation, Universiti Malaysia Sabah, Jalan UMS, Kota Kinabalu 88400, Sabah, Malaysia; 3 Enzyme and Microbial Technology Research Center, Faculty of Biotechnology and Biomolecular Sciences, Universiti Putra Malaysia, UPM Serdang 43400, Malaysia; suriana@upm.edu.my; 4 Department of Microbiology, Faculty of Biotechnology and Biomolecular Sciences, Universiti Putra Malaysia, UPM Serdang 43400, Malaysia

**Keywords:** farnesol, nerolidol, *Polygonum minus*, sesquiterpene synthase

## Abstract

*Polygonum minus* (syn. *Persicaria minor*) is a herbal plant that is well known for producing sesquiterpenes, which contribute to its flavour and fragrance. This study describes the cloning and functional characterisation of *Pm*STPS1 and *Pm*STPS2, two sesquiterpene synthase genes that were identified from *P. minus* transcriptome data mining. The full-length sequences of the *Pm*STPS1 and *Pm*STPS2 genes were expressed in the *E. coli* pQE-2 expression vector. The sizes of *Pm*STPS1 and *Pm*STPS2 were 1098 bp and 1967 bp, respectively, with open reading frames (ORF) of 1047 and 1695 bp and encoding polypeptides of 348 and 564 amino acids, respectively. The proteins consist of three conserved motifs, namely, Asp-rich substrate binding (DDxxD), metal binding residues (NSE/DTE), and cytoplasmic ER retention (RxR), as well as the terpene synthase family N-terminal domain and C-terminal metal-binding domain. From the in vitro enzyme assays, using the farnesyl pyrophosphate (FPP) substrate, the *Pm*STPS1 enzyme produced multiple acyclic sesquiterpenes of β-farnesene, α-farnesene, and farnesol, while the *Pm*STPS2 enzyme produced an additional nerolidol as a final product. The results confirmed the roles of *Pm*STPS1 and *Pm*STPS2 in the biosynthesis pathway of *P. minus*, to produce aromatic sesquiterpenes.

## 1. Introduction

Over the last 25 years, nearly 65,000 chemical structures of terpenoids have been discovered, making terpenoids the class of natural products with the greatest structural diversity [1,2]. Terpenoids are involved in a variety of important functions in regulating plant growth (especially for terpenoid lactones) and play an ecological role in attracting pollinators [3]. Terpenoids are grouped into different classes based on the number of 5-carbon building blocks [4,5,6,7]. All terpenoids are derived from the common phosphorylated five-carbon (C5) building units, isopentenyl diphosphate (IPP) and dimethylallyl diphosphate (DMAPP) [8]. There are two major pathways involved in the biosynthesis of terpenoids, namely, the mevalonate (MVA) pathway, which is primarily found in eukaryotes, and the methylerythritol phosphate (MEP) pathway (non-mevalonate pathway), which is primarily found in prokaryotes and plant chloroplasts [9,10,11]. For sesquiterpene biosynthesis, IPP and DMAPP undergo condensation to form farnesyl pyrophosphate (FPP), which is catalysed by the enzyme FPP synthase. The resulting linear FPP undergoes electrophilic cyclisation and rearrangement to form acyclic and non-acyclic sesquiterpenes, based on the sesquiterpene synthase enzyme reactions [12,13]. 

In particular, monoterpenes and sesquiterpenes are commonly present in plant essential oils, which are widely utilised for commercial purposes, such as fragrances, cosmetics, pharmaceuticals, medicine, biofuel precursors, and industrial materials [14,15]. To date, over 7000 sesquiterpenes (C15) with different hydrocarbon skeletons and stereo-chemically structures have been reported [16]. Studies on naturally derived 15-carbon terpenoids in aromatic herbal plants as anti-malarial, anti-microbial, and insecticidal agents have also increased over the last several years [17]. Hence, many sesquiterpenes are founds as major components of fruits and vegetables, floral scents, and essential oils of herbs. In previous studies, a number of genes encoding the sesquiterpene enzymes, which control the key steps of secondary metabolic pathways, have been extensively cloned and characterized from a number of herbal plant species. For example, squalene synthase from *Tripterygium wilfordii* [18], germacrene A synthase from *Achillea millefolium* [19], drimenol synthase from *Valeriana officinalis* [20], E-E-farnesol synthase and α-bisabolene synthase from *Gingko biloba* [21], α-humulene synthase from *Zingiber zerumbet* Smith [22], (+)-epi-α-bisabolol synthase from *Lippia dulcis* [23], β-caryophyllene synthase from *Ocimum basilicum* L. [24], α-humulene synthase from *Zingiber zerumbate* Smith [25], and Germacrene D synthase from *Zingiber officinale* [26]. 

*Polygonum minus* (syn. *Persicaria minor*) is a herbal plant that originated from Southeast Asia and belongs to the Polygonaceae family. In Malaysia, *P. minus* is locally known as a ‘kesum’, and is commonly used as a food additive and flavouring agent. Many studies have been carried out because of the popularity of the *P. minus* as a potential medicinal plant with high antioxidant and antimicrobial activities and strong anti-inflammatory properties [27,28,29,30]. *P. minus* is an economically important herbal plant because of its essential oils, which cause it to emit a strong scent from a simple mixture of terpenoid hydrocarbons (monoterpenoids and sesquiterpenoids), including β-farnesene, α-farnesene, nerolidol, farnesol, caryophyllene, α-bergamotene, and drimenol [31,32]. Other characteristic components that have been identified from *P. minus* include aldehydes (decanal and dodecanal), esters, and organic acids [33]. Moreover, recent studies have revealed that the sesquiterpenes compounds are the main contributors to the characteristic fragrance of this plant [34]. These studies have shown the potential for developing *P. minus* as a resource to produce natural products. While many structurally diverse secondary metabolites, especially terpenoid compounds, have been identified in *P. minus*, its biosynthesis of major constituents remains unclear because of the limited genomic information that is available for this plant. Therefore, this plant is investigated for the isolation and characterisation of novel sesquiterpene synthase genes. Additionally, there is not much work on sesquiterpene synthase from *P. minus*, especially at the genetic level, as it is still very scarce. 

In previous studies, several works on sesquiterpene synthases were identified. The first *P. minus* putative sesquiterpene synthase gene was cloned and expressed in *E. coli* systems [35]. Song et al. [36] successfully overexpressed a sesquiterpene synthase, *Pm*STS, in metabolically engineered gram-positive bacteria, *Lactococcus lactis*, with the MVA pathway. Then, a structural study was performed that demonstrated an active catalytic site with the same gene sequence [37]. The encoded enzyme was named β-sesquiphellandrene synthase, based on the principal product that was formed. In a subsequent study, the influence of jasmonic acid treatment on the expression level of the *Persicaria minor* sesquiterpene synthase (*Pm*SS) gene was reported [38]. Until very recently, the purification and overexpression of *P. minor* sesquiterpene synthase encoded as *Pm*STS recombinant protein in pET28b vector, using the *E. coli* BL21 (DE3) strain, were reported [39]. All of the studies on *P. minus* that have been mentioned above were based on the same sesquiterpene synthase. In order to understand the terpenoid metabolism of *P. minus*, the potential gene for terpene synthases must be isolated and studied. Therefore, in this study, we described the cDNAs isolation and characterisation of two new sesquiterpene synthases (STPS) that were responsible for the formation of two key aromatic compounds, which made substantial contributions to the flavour and fragrance of *P. minus* essential oil. The results could have provided a foundation for the further exploration of gene function in *P. minus*, and helped to reveal the regulation of terpenoid biosynthesis.

## 2. Results

### 2.1. Screening and Isolation of Sesquiterpene Synthase Gene from P. minus

Two new candidates of sesquiterpene synthase genes, *Pm*STPS1 (comp62410_co_seq6) and *Pm*STPS2 (comp47018_c0_seq1), were successfully identified through the sequence analysis of *P. minus* transcriptome [40]. The 1098 bp *Pm*STPS1 transcript contained an open reading frame (ORF) of 1047 bp, encoding 348 amino acids with a calculated molecular mass of 40.9 kDa and an isoelectric point (pI) of 6. 15 (Figure S1). The ORF of *Pm*STPS1 started from the nucleotide position at 25 and ended at position 1071. The deduced amino acid sequence of *Pm*STPS1 (GenBank accession no. MG921605) showed no signal peptide. A ProtParam analysis of the predicted amino acid sequence of *Pm*STPS1 revealed 47 negatively charged residues (Asp and Glu) and 52 positively charged residues (Arg and Lys), which represented the aliphatic index of this protein. This was a positive factor for the increased thermostability of the globular protein. The second sesquiterpene synthase transcript, *Pm*STPS2 (GenBank accession No: MG921606), was 1974 bp long and had an ORF of 1695, which encoded a polypeptide of 564 amino acids (Figure S2). The ORF of *Pm*STPS2 started from the nucleotide position at 97 and ended at position 1798. The calculated molecular mass of the mature protein was approximately 65.96 kDa, with a predicted pI of 5.75. A ProtParam analysis of the predicted amino acid sequence of *Pm*STPS2 identified 83 negatively charged residues (Asp and Glu) and 70 positively charged residues (Arg and Lys).

Based on the BLASTx analysis, the predicted amino acid sequences of *Pm*STPS1 (Table 1) and *Pm*STPS2 (Table 2) had the closest hit to drimenol synthase from *Persicaria hydropiper*, with a 96% and 46% identity, respectively. The predicted amino acid sequence of *Pm*STPS2 was consistent with those of other sesquiterpene synthases encoding proteins of 550–580 amino acids, with molecular weights of 60–70 kDa. Conversely, the length of *Pm*STPS1, with only 348 amino acids, was much shorter than that of the other sesquiterpene synthases. Therefore, only *Pm*STPS2 met the range of other reported plant terpene synthases [21,41,42,43,44,45].

The presence of the conserved domains in the *Pm*STPS1 and *Pm*STPS2 proteins was consistent with and similar to that of the other terpene synthase features. The terpene synthase family N-terminal domain (PF01397) and the synthase family C-terminal metal-binding domain (PF03936) contained highly conserved aspartate-rich motifs (DDxxD), which were essential for enzyme-substrate binding and catalytic function. The first aspartate-rich motif played a role in the determination of the chain length for the resulting prenyl pyrophosphate. 

**Figure 1:**
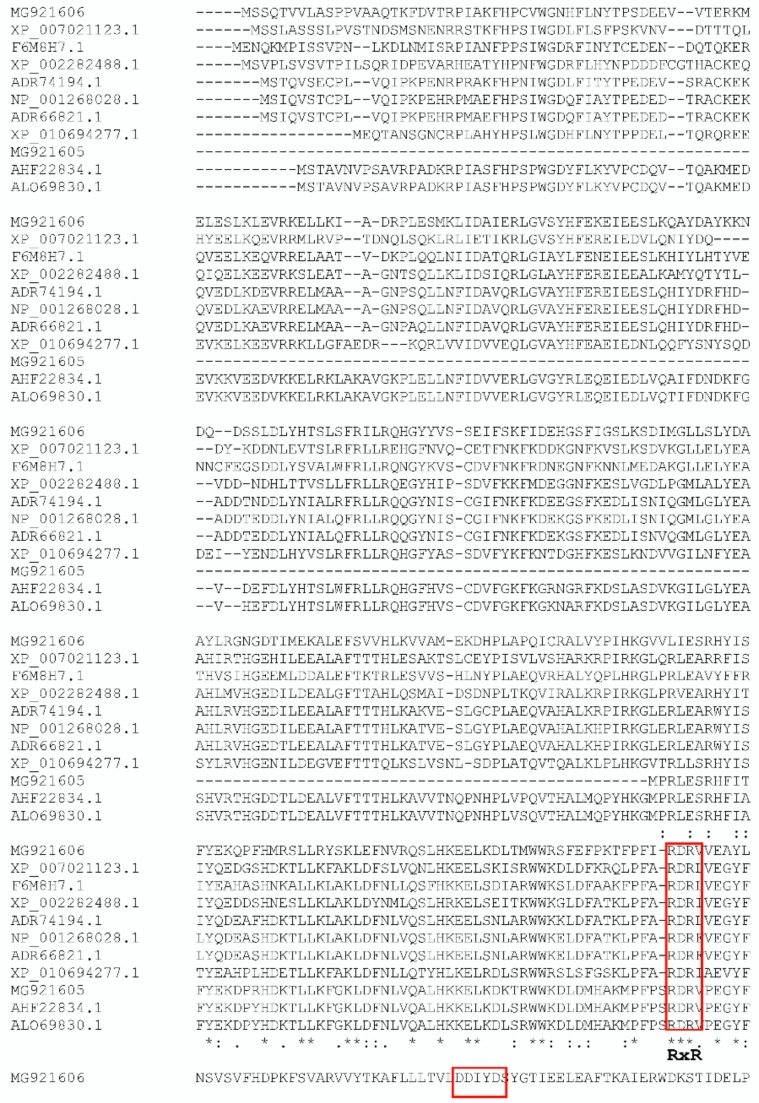

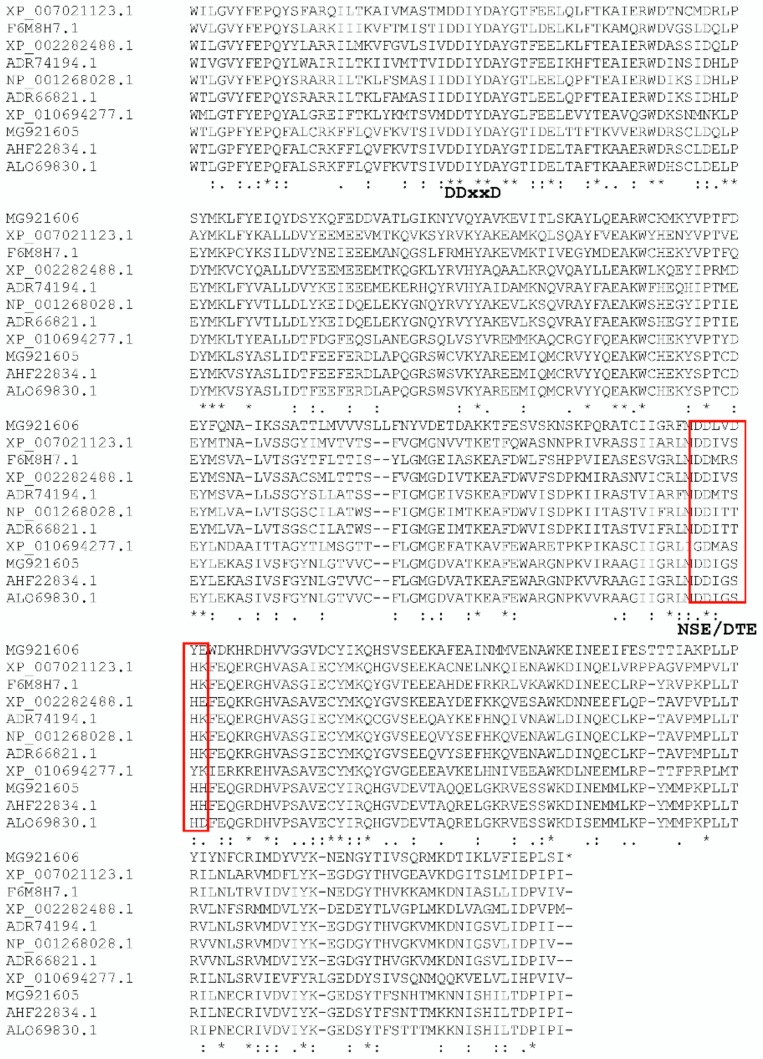
Comparison of the deduced amino acid sequence of *Pm*STPS1 and *Pm*STSP2, with other terpene synthase sequences of the highest sequence similarity. *Polygonum minus* sesquiterpene synthase 1 (MG921605); *Polygonum minus* sesquiterpene synthase 2 (MG921606); *Persicaria hydropiper* drimenol synthase (AHF22834.1); *Persicaria minor* putative sesquiterpene synthase (ALO69830.1); *Theobroma cacao* Delta-cadinene synthase isozyme A (XP_00702113.1); *Beta vulgaris* subs. vulgaris probable sesquiterpene synthase (XP_010694277.1); *Santalum murrayanum* probable sesquiterpene synthase (F6M8H1); *Vitis vinifera* (−)-Germacrene D synthase (XP_002282488.1); *Vitis vinifera* Valencene synthase (NP_001268028.1); and *Vitis vinifera* Germacrene A synthase (ADR61821.1). Amino acid residues conserved in all of the genes are marked with asterisk [*]. Amino acid residues conserved in four or five genes are indicated by double dots [:]. The universally conserved DDxxD, RxR motifs, and NSE/DTE are highlighted in boxes.

Based on the multiple sequence alignment of *Pm*STPS1 (Figure 1), several conserved motifs that were found in typical terpene synthases were identified, including the DDxxD (residue 100–104) and NSE/DTE (residue 245–253) motifs. The DDxxD and NSE/DTE motifs flanked the entrance of the active site. In addition to these motifs, there was a highly conserved arginine-rich RxR motif, which was involved in the complexing of the diphosphate group, after the ionisation of FPP [18,39]. The RxR motif was located at 45 amino acids, upstream of the first DDxxD motif. For *Pm*STPS2, the conserved arginine-rich (RxR) region at amino acid position 278–281 was conserved in all of the terpene synthases [19]. Moreover, the aspartate-rich motif of DDxxD, which might have been the Mg^2+^ binding site, was located at position 314–318 of the amino acid sequence. Another metal binding motif, the NSE/DTE motif, was detected at amino acid position 461–467 (Figure 1). The less conserved motif NSE/DTE, apparently evolved from a second motif that was conserved in prenyl transferase. In general, these motifs were located on the opposite sides of the active site [6,7]. The metal binding residues appeared as NSE in most microbial and fungal cyclases and as DTE in most plant cyclases. 

### 2.2. Phylogenetic Analysis of P. minus Sesquiterpene Synthase (PmSTPS)

**Figure 2:**
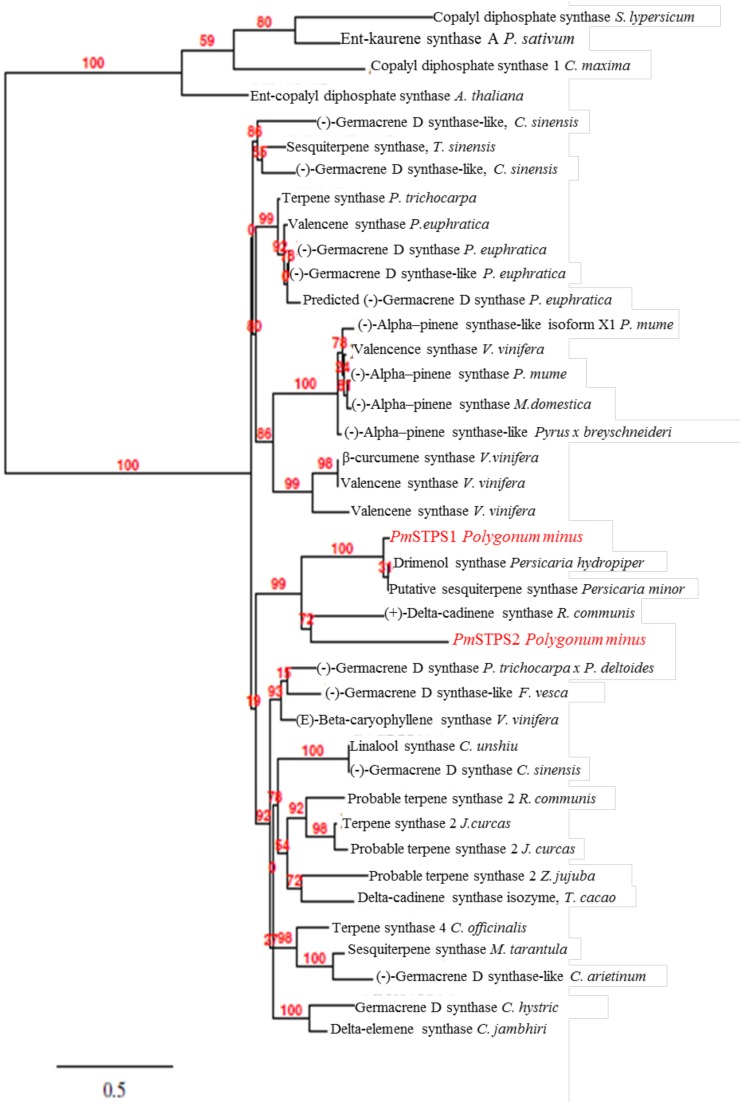
Phylogenetic tree of *Pm*STPS1 and *Pm*STPS2 protein sequences with amino acid sequences, with selected terpene synthases from other plants. The alignment was performed using the Clustal Omega algorithm. The tree was built using the neighbor-joining method and 1000 replicates for bootstrapping. The numbers indicated are the actual bootstrap values of the branches.

The *Pm*STPS1 and *Pm*STPS2 amino acid sequences were aligned and compared with other flowering plant terpene synthase sequences, using Clustal Omega (Figure 2), and they showed a low sequence similarity (42.94%). The phylogenetic analysis showed a particularly close relationship between the *Pm*STPS1 and *Pm*STPS2 amino acid sequences. The *Pm*STPS1 was clustered in the same clade with sesquiterpene synthase (*Pm*STS) from *Persicaria minor* and drimenol synthase from *Persicaria* hydropiper. The results showed that the *Pm*STPS1 from *P. minus* was grouped into a single clade with a 43.04% identity, which suggested a monophyletic origin of the gene. Additionally, *Pm*STPS2 was placed in the same clade with (+) delta-cadinene synthase from *Ricinus communis*. Moreover, *Pm*STPS1 and *Pm*STPS2 were grouped together with the terpene synthases from the Santalum and *Vitis vinifera* species. Multiple sequence alignments of *Pm*STPS1 and *Pm*STPS2 amino acid sequences, with sesquiterpenes from other plants species, showed a high sequence similarity (42–96%).

### 2.3. Expression of PmSTPS1 and PmSTPS2 in E. coli

For the analysis of the protein expression, recombinant bacterial strains harbouring pQE2 in *E. coli* M15 with *Pm*STPS1 and *Pm*STPS2 were compared with those harbouring the control empty pQE2 vector. The cells were harvested at different times (1, 3, and 5 h) post-induction. After sonication and centrifugation of the bacteria, soluble and insoluble crude fractions were separated with 10% SDS-PAGE. The SDS-PAGE analysis (Figure S3) showed unclear corresponding protein bands at the expected size at different post-induction times, as well as in the control sample. However, Western Blotting (Figure S4) confirmed the correct size of recombinant proteins. There was no band was observed in the control sample as expected. Correct protein sizes of 40.9 and 65.9 kDa were obtained for *Pm*STPS1 and *Pm*STPS2, respectively. From these findings, the recombinant *Pm*STPS1 and *Pm*STPS2 proteins from *P. minus* were successfully expressed in *E. coli*, and the activities of these enzymes were further investigated via enzymatic assays.

### 2.4. Identification of PmSTPS1 and PmSTPS2 Assay Products

**Figure 3:**
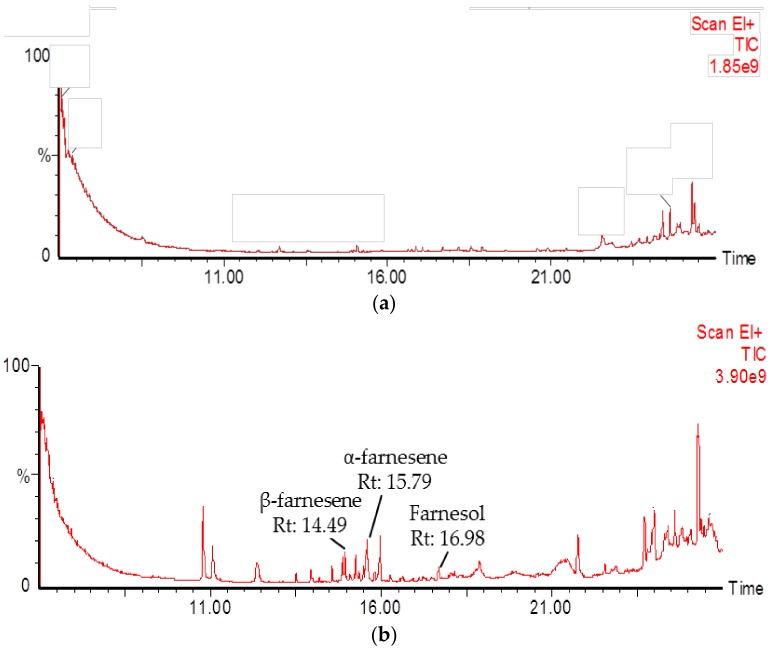

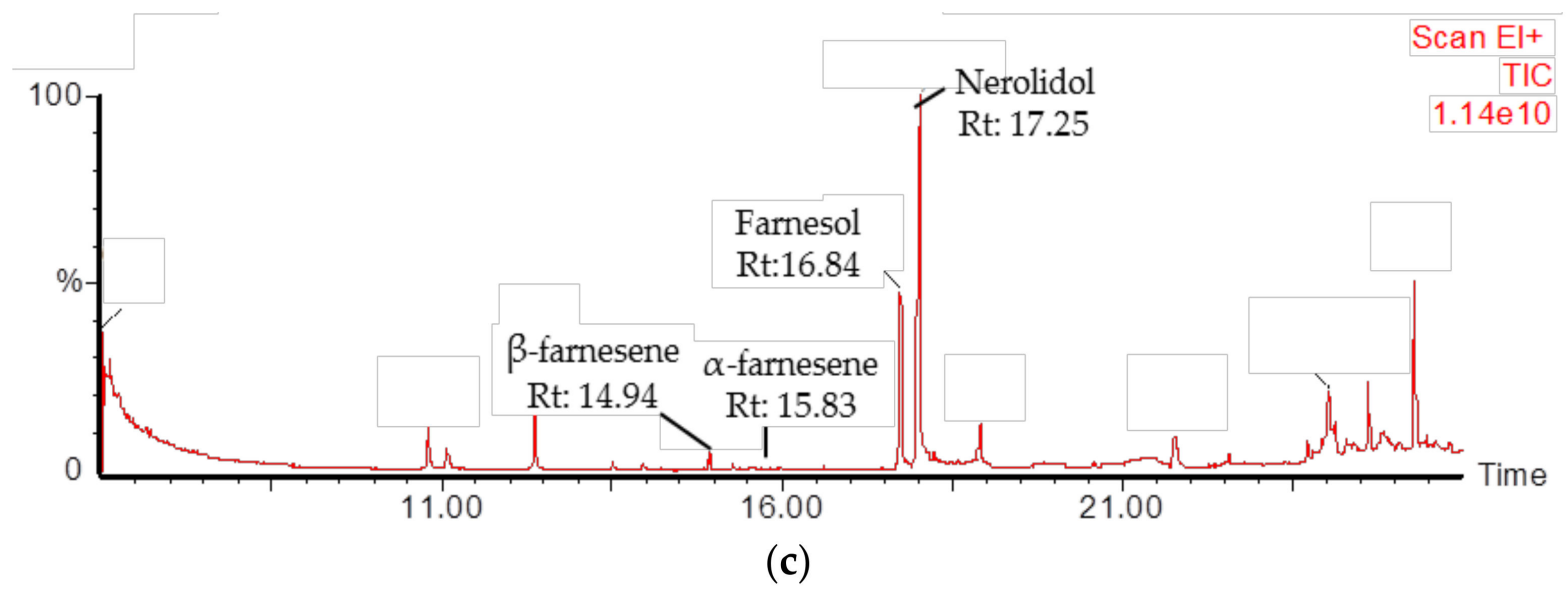
GC-MS chromatogram of products formed by crude *Pm*STPS1 and *Pm*STPS2 protein at different retention times (RT). (**a**) The chromatogram of control used consisted of M15 *E. coli* host harboring empty pQE-2 taqzyme plasmid; (**b**) *Pm*STPS1 (**c**); and *Pm*STPS2.

A functional characterisation of the *Pm*STPS1 and *Pm*STPS2 genes was performed by an in vitro enzyme assay of the recombinant proteins. In this crude protein assay, the *E. coli* strain harbouring empty pQE-2 vector was used as the control strain. A GC-MS analysis showed that *Pm*STPS1 and *Pm*STPS2 produced β-farnesene, α-farnesene, and farnesol as the final products. Additionally, *Pm*STPS2 also produced nerolidol. For *Pm*STPS1, the products formed were β-farnesene (14.49 min), α-farnesene (15.79 min), and farnesol (16.98 min). Additionally, the principle products from *Pm*STPS2 enzyme were β-farnesene (14.49 min), α-farnesene (15.83 min), farnesol (16.84 min), and nerolidol (17.25 min). Based on the GC-MS analysis, both extracts from *Pm*STPS1 and *Pm*STPS2 showed multiple peaks for corresponding sesquiterpene products, compared with no peaks observed in the control sample, which did not exhibit any major products, although exogenous substrates were added (Figure 3). These findings therefore demonstrated the successful production of sesquiterpenes in the recombinant *E. coli* strains overexpressing *Pm*SPTS1 and *Pm*STPS2 enzymes, respectively.

All of the peaks were further confirmed by comparison with NIST and Wiley libraries, mass spectra, authentic sesquiterpene standards, and control (Figure S5). Interestingly, although the sizes of *Pm*STPS1 and *Pm*STPS2 were different, the two enzymes were capable of producing similar sesquiterpene products, β-farnesene, α-farnesene, and farnesol (Figure 3), but they did so at different levels. *Pm*STPS1 successfully converted the precursor FPP to produce 9.50% β-farnesene as the main product, followed by 8.86% α-farnesene, and 5.08% farnesol. *Pm*STPS2 showed synthesises nerolidol (48.33%) as a major product, followed by farnesol (15.30%), β-farnesene (5.07%), and α-farnesene (2.76%). Although the (E,E)-farnesyl pyrophosphate (FPP) substrate was added to the enzymatic assay, the control pQE-2 sample did not produce any significant products, indicating that endogenous metabolites did not affect the protein expression and analysis in this study.

## 3. Discussion

In this study, we provided the first cloning and functional characterisation of *Pm*STPS1 encoding a putative β-farnesene synthase, and *Pm*STPS2 encoding a putative nerolidol synthase in *P. minus*. A sequence comparison between the *Pm*STPS1 and *Pm*STPS2 indicated that the two enzymes had different protein and nucleotide sequences. However, both of the enzymes were structurally similar to other plant sesquiterpene synthases and contained all of the conserved motifs, including DDxxD, RXR, and NSE/EDTA, which were important for terpene synthase functionality [5,45,46]. Based on the phylogenetic analysis, *Pm*STPS1 and *Pm*STPS2 clustered on the same group with four distinct sesquiterpene synthases.

In addition, the unexpected band in the upper layer (Figure S4) might have been caused by protease aggregation, according [47]. As shown in several recent studies, the protein sizes of a few plants sesquiterpene synthases showed a nearly similar molecular weight, with *Pm*STPS2 within the range of 60–70 kDa [24,48,49]. In general, terpene synthase could be classified into monoterpene, sesquiterpene, and diterpene synthase, with 550–860 amino acids encoding a 50–100 kDa protein [4,50]. As a result of the absence of the signal peptide sequence with 50–70 amino acids, the size of sesquiterpene synthases were typically smaller than those of the monoterpenes and diterpenes [51]. Ee et al. [37] also reported that the protein size of the *P. minus* β-sesquiphelandrene synthase was 65.1 kDa. Additionally, studies on β-caryophyllene synthase, that were encoded by OkBCS (GenBank accession no. KP226502) from *Ocimum kilimandscharicum* Gürke, showed a molecular weight of 63.6 kDa [24]. Until recently, no short sesquiterpene synthase sequence was characterised as a *Pm*STPS1. However, the short-chain length of this enzyme could be associated with several prenyltransferase (PT) enzymes. Based on previous findings, two prenyltranferases, Santalum farnesyl diphosphate synthase (SaFDS) and Hedychium farnesyl pyrophosphate synthase (HcFPPs), comprising 1029 and 1068 bp nucleotide sequences and encoding polypeptides of 343 and 356 amino acids, respectively, were reported [3,52]. 

Many terpene synthases (TPSs) had the ability to synthesise one or multiple products from a single substrate, regardless of whether it was farnesyl pyrophosphate (FPP) or geranyl pyrophosphate (GPP) [53,54]. In addition, the sesquiterpene synthases from different plant species produced more than one product [25,55,56]. Interestingly, although the sizes of *Pm*STPS1 and *Pm*STPS2 were different, the two enzymes produced similar sesquiterpene products (β-farnesene, α-farnesene, and farnesol, Figure 3), albeit at different percentages. Both *Pm*STPS1 and *Pm*STSP2 could catalyse the formation of β-farnesene, α-farnesene, nerolidol, and farnesol, whose functions were different from those of the previous STPS enzymes characterised in *P. minus* [36,37,38,39]. In addition, the enzymes demonstrated an inherent capacity for TPS enzymes to evolve different products and substrate specificities [57]. Moreover, the main factor of sesquiterpene diversity was the large number of different sesquiterpene synthases expressed in plants and the ability of some sesquiterpene synthases to form multiple products from a single FPP substrate [58]. 

Based on previous chemical profiling studies of *P. minus* essential oils from hydro-distillation extraction, low levels of nerolidol and farnesol were detected at 0.24% and 0.14%, respectively [34]. A similar finding was reported, which indicaed that the percentage of β-farnesene and α-farnesene compounds were also found at 0.92% and 0.82%, respectively [31,32,34]. The percentage of terpenes that were obtained directly from the GC-MS analysis of *P. minus* leaf essential oil was lower compared with the products that were produced by the enzymatic assay of crude protein *Pm*STPS1 and *Pm*STPS2. The variation in composition could have been because of the variable amounts of sesquiterpenes that were produced in the plants, depending on environmental factors. Nevertheless, the sesquiterpenoids that were produced by in vitro assay potentially contributed to the plant fragrance, because most of the acyclic sesquiterpenes compounds, namely, farnesene, nerolidol, and farnesol, were previously reported in various plant essential oils [21,59,60]. These compounds could be potentially commercialised as fragrances, flavouring agents, or pharmaceutical products. In addition, farnesene is an important compound for diesel and jet fuels [61]. Understanding the physiological and ecological roles of plant volatile sesquiterpenes has been challenging. Several sesquiterpenes compounds might have acted as defense chemicals against biological stresses. For instance, E,E-α-farnesene was reported to have potential for use as an alarm pheromone in the control of aphid pests [62]. Furthermore, the existence of farnesol in *P. minus* essential oil could have been related to the biosynthetic pathway of juvenile hormone (JH) III [63]. Nerolidol was not only used in cosmetics and non-cosmetic products [64], but was also proven to possess pharmacological and biological activities [65,66]. Therefore, the advantages of nerolidol have made it a promising drug candidate for industrial production [67,68]. 

## 4. Materials and Methods 

### 4.1. Plant Material 

*P. minus* plants was grown in an experimental plot at Universiti Kebangsaan Malaysia (UKM) under natural light and environmental conditions. The samples were originally collected from Ulu Yam, Selangor, Malaysia (UY; 3°16′14.63′′ N, 101°41′11.32′′ E), and were identified using ITS sequences [69]. The voucher specimens were deposited at the UKM herbarium. Leaf samples from *P. minus* plants were harvested in the morning, between 8 to 9 am, frozen in liquid nitrogen, and stored at −80 °C for RNA extraction.

### 4.2. RNA Isolation and cDNA Synthesis

Total RNA was isolated and extracted as it was previously reported [70]. The quantity, purity, and integrity of the RNA were determined using standard methods. Three micrograms of RNA were reverse transcribed into cDNA using the Onetaq^®^One-step RT-PCR kit (New England Biolabs, Ipswich, MA, USA), according to manufacturer’s instructions. 

### 4.3. Candidate Gene Selection and Isolation of Full-Length PmSTPS1 and PmSTPS2

The candidate gene selection was achieved by mining the *P. minus* transcriptome data [40] for transcripts that were related to the sesquiterpene biosynthetic pathway. The assembled transcripts were classified as sesquiterpene synthase, based on homology search, and the terpene synthases were selected and fully sequenced prior to further analysis. Two new *P. minus* sesquiterpene synthase (*Pm*STPS) candidate genes were identified. The predicted ORFs for *Pm*STPS1 (GenBank accession no: MG921605) and *Pm*STPS2 (GenBank accession no: MG921606) were amplified by PCR, using the Q5 High-Fidelity DNA Polymerase (NewEngland Biolabs, Ipswich, MA, USA). The cDNA-gene specific PCR primers were *Pm*STPS1_F (5’-AAAGGTACCATGCCAA GGCTCG-3′) and *Pm*STPS1_R (5′-TTTGTCGACAATCGGAATGGGAT-3′); *Pm*STPS2_F (5′-AAAGGTACCATGTCAT

CCCAAA-3′), and *Pm*STPS2_R (5′-TTTAAGCTTAATGGAGAGAGGTT-3′) were synthesized to amplify the 5′ end and 3′ end, respectively.

The PCR reaction mixture contained 1× reaction buffer, 2 mM MgCl_2_, 0.2 mM dNTPs, 5 units of Taq polymerase (Promega, Madison, WI, USA), 0.5 µM of forward and reverse primers, and 20 ng of template cDNA. The reaction was performed under the following conditions: pre-denaturation at 98 °C for 30 s, followed by 32 cycles of 98 °C for 10 s, 60 °C for 10 s, and 72 °C for 20 s, with a final extension at 72 °C for 2 min. The amplicons were digested with *Kpn*I/*Sal*I and *Kpn*I/*Hind*III before being cloned into pUC19 and pUC57-Kan cloning vectors, respectively. The ligation mixtures were transformed into *E. coli* top 10 competent cells, before sub-cloning into the pQE-2 plasmid. The positive transformants were screened on LB agar that were supplemented with 100 µg/mL ampicillin, 20 µg/mL X-gal, and 0.1 mM IPTG (ThermoFisher Scientific, Waltham, MA, USA). The recombinant plasmids pQE-2:*Pm*STPS1 and pQE-2:*Pm*STPS2 were then transformed into the *E. coli* M15 competent cells and the transformants were selected on LB agar that was supplemented with 50 µg/mL kanamycin. The positive transformants were confirmed by colony PCR, and the gene sequences were verified via DNA sequencing (First BASE Laboratories, Seri Kembangan, Selangor, Malaysia).

### 4.4. Full-Length cDNA Sequence Analysis and Phylogenetic Tree Construction

The ORF for *Pm*STPS1 and *Pm*STPS2 were predicted using the ORF finder program (http://www.ncbi.nlm.nih.gov) and were subjected to BLASTX and BLASTP analyses. Multiple sequence alignment was achieved using the Clustal Omega pairwise alignment algorithm. Verification of the cDNA sequence, including the amino acid sequence, theoretical isoelectric point (pI), and predicted molecular weight (MW) of the analyses, was performed using ExPASy Proteomic tools (http://www.cn.expasy.org/tools/protscale.html). The physical and chemical characteristics of all of the deduced amino acid sequences were analysed by the ProtParam tool (http://web.expasy.org/program/). The signal peptide targeting location of the deduced proteins was predicted using the SignalP method (http://www.cbs.dtu.dk/services/SignalP) and ChloroP program (http://www.cbs.dtu.dk/services/ChloroP/). A protein domain analysis was performed using the SMART (Simple Modular Architectural Research Tool) database (http://smart.embl-heidelberg.de/).

### 4.5. Phylogenetic Analysis

Phylogenetic and molecular evolutionary analyses of the amino acid sequences of the *Pm*STPS1 and *Pm*STPS2 from different plant species were constructed using the default parameters of PhyML software, which were available at Phylogeny.fr web services (www.phylogeny.fr/version2_cgi/simple phylogeny.cgi) [71]. PhyML was employed to construct a phylogenetic tree, by generating multiple alignments through the neighbour-joining computational method. 

### 4.6. Expression of PmSTPS1 and PmSTPS2 in E. coli

A single colony of recombinant *E. coli* M15 cells harbouring pQE-2:*Pm*STPS1, pQE2:*Pm*STPS2, and empty pQE-2 (as a negative control) were inoculated into 10 mL of an LB medium containing kanamycin (50 µg/mL), and were grown overnight at 37 °C. Approximately 2 mL of the cultures were added to 200 mL of fresh LB, which contained 50 µg/mL kanamycin. The cultures were induced with 0.5 mM IPTG at OD600 ~0.5. The cultures were incubated for 1, 3, and 5 h at 37 °C, and were then harvested by centrifugation at 4000× *g* for 30 min at 4 °C. Subsequently, the bacteria were resuspended in 100 mL of 25 mM sodium phosphate buffer, pH 7.5, containing 0.5 M Tris–HCl, 5% glycerol, 1 mM dithiothreitol (DTT), 10 mM MgCl_2_, 1 mM MnCl_2_, pH 7.5, and 1 mM lysozyme (Sigma-Aldrich, St. Louis, MI, USA) [20]). The cells were sonicated for 2 min at 5 s pulses, with 5 s between the pulses on ice, using the Sonic Dismembrator Model 100 (Fisher Scientific, Hampton, NH, USA). The cell lysate was then centrifuged at 10,000× *g* for 30 min at 4 °C.

### 4.7. Enzyme Assay

A standard assay was done according to a previous method, with slight modifications. Standard assays were performed in 2.5 mL glass GC vials containing 200 µg of crude protein mixed with 50 mM Tris (pH 7.5), 10 mM MgCl_2_, and 100 µM of (E,E)-farnesyl pyrophosphate (FPP) (Sigma-Aldrich, St. Louis, MI, USA). The reaction mixture with a total volume of 200 µL was vortexed, overlaid with 500 mL hexane, and incubated at 30 °C for 2 h. The hexane phase was concentrated to 200 mL by passing N_2_ at the opening of the tube and was then further used for the GC-MS analysis. 

### 4.8. Detection of Sesquiterpenes Using GC-MS

The samples were analysed using a Clarus 600 GC-MS (PerkinElmer Inc., Waltham, MA, USA) that was equipped with a capillary column (Elite-5 30 m × 0.25 mm, film thickness 0.25 µm). The GC was operated at a flow rate of 2 mL/min, and the mass selector detector (MSD) was operated at 70 eV. Splitless injections (1.5 µL) were performed with an injector temperature of 250 °C. The GC system was programmed with an initial oven temperature of 50 °C (5 min hold), which was then increased to 180 °C at 10 °C/min (4 min hold), followed by a 100 °C/min ramp at 240 °C (1 min hold). A solvent delay of 8.5 min was allowed before the acquisition of the MS data. The MS system was operated in selected ion monitoring (SIM) mode to scan for the molecular ions at product peaks, which were quantified by the integration of peak areas with library search, using the NIST library [72]. 

## 5. Conclusions

In summary, two new sesquiterpene synthases, *Pm*STPS1 and *Pm*STPS2, which were identified from *P. minus* leaf transcriptomics analysis, were cloned and characterised. Both of the enzymes produced industrially important acyclic sesquiterpenes, β-farnesene, α-farnesene, and farnesol. *Pm*STPS2 also produced nerolidol as the major product from FPP conversion. This study demonstrated the production of *P. minus* characteristic fragrance-related sesquiterpenes, by both *Pm*STPS1 and *Pm*STP, as well as the potential of further metabolic engineering in *E. coli*, using *Pm*STPS2 for the microbial production of nerolidol.

## Figures and Tables

**Table 1:** BLASTx analysis of *Pm*STPS1 with the NCBI protein database.

Description	Organism	Score	E-value	Identity (%)	Accession
Drimenol synthase	*Persicaria hydropiper*	678	0.0	96	AHF2284.1
Putative sesquiterpene synthase	*Persicaria minor*	664	0.0	95	ALO69830.1
Delta-cadinene synthase isozyme A	*Theobroma cacao*	393	5 × 10^−129^	54	XP_007021123.1
Predicted: probable sesquiterpene synthase	*Beta vulgaris* subsp. *vulgaris*	389	3 × 10^−127^	54	XP_010694277.1
Predicted: probable terpene synthase 2	*Rocinus communis*	381	3 × 10^−124^	52	XP_002523635.1
Probable sesquiterpene synthase	*Santalum murrayanum*	379	1 × 10^−123^	54	F6M8H1
(−)-germacrene D synthase-like isoform X2	*Citrus sinensis*	379	1 × 10^−123^	52	XP_015384843.1

**Table 2:** BLASTx analysis of *Pm*STPS2 with the NCBI protein database.

Description	Organism	Score	E-value	Identity (%)	Accession
Drimenol synthase	*Persicaria hydropiper*	492	3 × 10^−164^	46	AHF2284.1
Probable sesquiterpene synthase	*Beta vulgaris* subsp. *vulgaris*	492	3 × 10^−164^	43	XP_010694277.1
Valencene synthase-like	*Vitis vinifera*	489	2 × 10^−163^	44	NP_001268028.1
Germacrene A synthase	*Vitis vinifera*	489	2 × 10^−163^	44	ADR66821.1
Putative sesquiterpene synthase	*Persicaria minor*	484	3 × 10^−161^	45	ALO69830.1
Predicted: (−)-germacrene D synthase	*Vitis vinifera*	479	4 × 10^−159^	42	XP_002282488.1
(E)-beta-caryophyllene synthase	*Vitis vinifera*	477	1 × 10^−158^	45	ADR74194.1

## Supplementary Materials

The following are available online, Figure S1: Nucleotide and predicted amino acid sequence of *Pm*SPTS1 and from *P. minus*; Figure S2: Nucleotide and predicted amino acid sequence of *Pm*STPS2 from *P. minus*.; Figure S3: SDS-PAGE analysis of recombinant pQE2_*Pm*STPS1 and pQE2-*Pm*STPS2 proteins is marked with red box and molecular mass markers are indicated; Figure S4: The expression analysis of *Pm*STPS1and *Pm*STPS2 in *E. coli* M15 after 0.5 mM IPTG induction at 1, 3, and 5 h; and Figure S5: Mass spectra of major three sesquiterpenes produced by recombinant *Pm*STPS1 and *Pm*STPS2 in comparison with the mass spectra from authentic standards.
